# A Comparison of Network-Based Methods for Drug Repurposing along with an Application to Human Complex Diseases

**DOI:** 10.3390/ijms23073703

**Published:** 2022-03-28

**Authors:** Giulia Fiscon, Federica Conte, Lorenzo Farina, Paola Paci

**Affiliations:** 1 Department of Computer, Control and Management Engineering “Antonio Ruberti” (DIAG), Sapienza University of Rome, Via Ariosto 25, 00185 Rome, Italy; fiscon@diag.uniroma1.it (G.F.); lorenzo.farina@uniroma1.it (L.F.); 2 Institute for Systems Analysis and Computer Science “Antonio Ruberti”, National Research Council, Via dei Taurini 19, 00185 Rome, Italy; federica.conte@iasi.cnr.it

**Keywords:** network medicine, drug repurposing, network theory

## Abstract

Drug repurposing strategy, proposing a therapeutic switching of already approved drugs with known medical indications to new therapeutic purposes, has been considered as an efficient approach to unveil novel drug candidates with new pharmacological activities, significantly reducing the cost and shortening the time of de novo drug discovery. Meaningful computational approaches for drug repurposing exploit the principles of the emerging field of Network Medicine, according to which human diseases can be interpreted as local perturbations of the human interactome network, where the molecular determinants of each disease (disease genes) are not randomly scattered, but co-localized in highly interconnected subnetworks (disease modules), whose perturbation is linked to the pathophenotype manifestation. By interpreting drug effects as local perturbations of the interactome, for a drug to be on-target effective against a specific disease or to cause off-target adverse effects, its targets should be in the nearby of disease-associated genes. Here, we used the network-based proximity measure to compute the distance between the drug module and the disease module in the human interactome by exploiting five different metrics (minimum, maximum, mean, median, mode), with the aim to compare different frameworks for highlighting putative repurposable drugs to treat complex human diseases, including malignant breast and prostate neoplasms, schizophrenia, and liver cirrhosis. Whilst the standard metric (that is the minimum) for the network-based proximity remained a valid tool for efficiently screening off-label drugs, we observed that the other implemented metrics specifically predicted further interesting drug candidates worthy of investigation for yielding a potentially significant clinical benefit.

## 1. Introduction

Drug repurposing is a drug development strategy used to identify novel uses for drugs approved by the US Food and Drug Administration (FDA) outside the scope of their original medical indication [1]. Establishing if an ‘old drug’ can be reused for new therapeutic purposes could represent a faster and cheaper alternative to the de novo drug discovery process that generally takes 2–3 billion dollars and 12–15 years to be completed (from production to approval, passing through the various phases of preclinical and clinical trials) [1]. In the development of meaningful computational approaches for drug repurposing, very promising insights comes from the newly emerging field of Network Medicine [2,3], which applies tools and concepts from network theory to elucidate the relation between perturbations on the molecular level and phenotypic disease manifestations. According to the Network Medicine paradigm, the efficacious treatment of complex diseases can come up only from the knowledge of the broader network context of the molecular determinants of diseases (named *disease genes*) in the human interactome (i.e., the cellular network of all physical molecular interactions) [4]. It is becoming increasingly clear that the *disease genes* have a high propensity to interact with each other and agglomerate in locally dense and topologically well-defined regions of the interactome known as *disease modules*, whose perturbations may contribute to the pathobiological phenotype [3,5,6,7,8,9,10,11]. Following the Network Medicine perspective, even the action of a drug can be interpreted as a local perturbation of the interactome, and thus, for a drug to be on-target effective against a specific disease or to cause off-target adverse effects, its target proteins should be within or in the immediate vicinity of the corresponding disease module. In recent years, several network-based approaches marrying this philosophy have been developed to aid the identification of the specific interactome neighborhood that is perturbed in a certain disease [12,13,14,15,16] and/or for the effect of a certain drug, and guide the search for therapeutic targets, identify comorbidities, as well as rapidly detect drug repurposing candidates [17,18,19,20,21,22,23,24]. In order to quantify the interplay between drug targets and disease-specific proteins in the human interactome, in [17], the authors used a network-based drug-disease proximity measure, which prioritized associations between drugs and diseases located in the same network neighborhoods based on the average shortest paths.

Here, we exploited the network-based drug-disease proximity measure proposed in [17] by using four different metrics (i.e., maximum, mean, median, mode) to compute the distance between the drug module and the disease module of four diseases of interest (i.e., liver cirrhosis, malignant breast neoplasm, schizophrenia, and prostate neoplasm). Then, we compared the obtained candidate drugs with respect to those ones obtained by using the standard metric (i.e., minimum) [17]. Our outcomes confirmed that the original network-based proximity metric remained a valid tool for screening off-label drugs, but we also observed that the additional here-implemented metrics specifically highlighted some interesting drug candidates, with clues of potential in silico-efficacy, which were thus worthy of further investigation. These results suggested that this network-based approach can be generalized to other diseases and drugs, and this is the reason why we published the R-code along with this study, freely available at https://github.com/giuliafiscon/GeneralizedProximity.git (accessed on 27 March 2022).

## 2. Results

We evaluated the extent to which a given drug could be repositioned to treat a given disease by exploiting the network-based proximity measure relying on the distance between drug modules and disease modules in the human interactome network.

In order to topologically quantify this distance, we used four different metrics (i.e., maximum, mean, median, mode) and then compared the results with the standard metric based on the average shortest paths between drug targets and disease genes. The study design is depicted in Figure 1. In particular, the input data of our analysis were the human interactome, the list of disease-associated genes, and the drug-targets interactions. In the present study, the human interactome was downloaded from Cheng and co-authors [17], which is an integrated version of 15 different databases of protein–protein interactions; disease-associated genes were downloaded from DisGeNET [25], which is a knowledge-based platform integrating and standardizing data about disease-associated genes and variants from multiple sources; and drug-target associations were obtained from DrugBank [26], which collects a huge amount of drug-related data, recently enabling the discovery and repurposing of a relevant number of existing drugs to treat rare and newly identified diseases [1,17]. We assembled target information for a total of 1222 FDA-approved drugs, and we applied our algorithm to four diseases with the highest number of disease-associated genes (i.e., liver cirrhosis, malignant neoplasm of breast, prostate neoplasm, and schizophrenia). The complete lists of the analyzed diseases and drugs are provided in Supplementary Table S1.

Following the Network Medicine principles, for a drug to be effective against a specific disease, its associated targets (drug module) and the disease-specific associated genes (disease module) should be nearby in the human interactome [17]. To quantify the vicinity between a given drug module *T* and a given disease module *S*, we used the network proximity measure *p* defined as:

$$p\left(T,S\right)\;=\frac{1}{\left\|T\right\|}\underset{t\epsilon T}{\sum }\underset{s\epsilon S}{f(}d(t,s)\;)$$

where *f* function refers to five different metrics, including the standard minimum measure [17] and the other here-proposed ones (i.e., maximum, mean, median, mode), which we implemented to summarize the distance between drug targets *t* in the drug module *T* and the disease genes *s* in the disease module *S*, and thus to prioritize the predicted off-label drug indications for a given disease. For each metric, we complemented the computation of the proximity measure with a measure of statistical significance (*p*-value) by applying a degree-preserving randomization procedure (see Section 4). Thus, we considered eligible candidates’ drugs to be repositioned for a given disease those drugs whose targets were nearby in the interactome to the disease-associated genes more than expected by chance (*p*-value ≤ 0.05).

The results obtained for each disease are summarized in Figure 2a, whereas the complete lists of candidate repurposable drugs predicted by each metric are reported in Supplementary Table S2.

Overall, computing the proximity values by using the minimum metric produced the largest number of statistically significant predicted drugs for each disease, while no compounds were predicted in a statistically significant way by using the maximum metric (Figure 2a). Yet, by retrieving from the Therapeutic Target Database (TTD) [27] the original medical indications for each predicted drug, we observed that the mode metric allowed to identify the highest percentage of predicted drugs with an already established indication, greater than 60% for all the four analyzed diseases (ranging from 60% for malignant breast neoplasm to 95% for prostate neoplasm), immediately followed by the minimum, median, and mean metrics with a percentage greater than 60% for 3 out of 4 analyzed diseases (Figure 2b and Supplementary Table S3).

### 2.1. In Silico Efficacy: Two Case Studies

In order to further investigate the repurposable drugs predicted by using each metric and pointing out those ones that could counteract the disease effect, we performed a gene set enrichment analysis (GSEA) for two case studies (i.e., malignant breast neoplasm and prostate neoplasm) detailed in the following sections.

#### 2.1.1. Malignant Breast Neoplasm

For studying the effect of eligible drugs on human breast cancer, we exploited:

(i) The drug-treated human breast adenocarcinoma cell line (i.e., MCF7) is available from the Connectivity Map (CMap) database as drug signature;
(ii) The differentially expressed genes for breast invasive carcinoma dataset are available from The Cancer Genome Atlas (TCGA) repository as disease signature (see Materials and Methods).

We also investigated the subtypes distribution of the cohort of TCGA breast cancer patients. In particular, by retrieving the clinical information, we obtained the HER2+/−, ER+/−, and PR+/− status for 77 (out of 113) patients, corresponding to the 68% of the total number of analyzed breast cancer patients. Among the 77 classified patients, we observed 81% (62/77) characterized by a less aggressive subtype (i.e., luminal A/B/B-like) and 19% (15/77) by a more aggressive subtype (i.e., HER2+ and triple negative) (Table 1). This observation strongly supports the usage of MCF7 cell line available from CMap, that is a poorly aggressive and non-invasive breast cancer cell line.

Then, we calculated a GSEA score as an indication of the possible counteraction of each drug to the gene expression perturbations caused by the breast cancer pathophenotype. In particular, we selected drugs whose signatures were negatively correlated with the breast cancer signature, according to the CMap query tool [28,29,30], as drugs able to have a potential treatment effect against genes that are a hallmark of breast cancer phenotype (see Section 4).

Overall, the GSEA analysis confirmed that the minimum metric specifically predicted the highest percentage equal to 22% of the candidate drugs with potential in silico efficacy able to counteract the disease effect (i.e., with GSEA score > 0), immediately followed by the mode metric with a percentage of GSEA confirmed drugs equal to 18%. (Figure 3, Supplementary Table S4—first sheet).

#### 2.1.2. Prostate Neoplasm

For studying the effect of eligible drugs on human prostate cancer, we used:

(i) The drug-treated human prostate adenocarcinoma cell line (i.e., PC3) from CMap database as drug signature;
(ii) The differentially expressed genes for prostate adenocarcinoma dataset available from TCGA repository as disease signatures (see Section 4).

Then, we calculated a GSEA score as an indication of the possible counteraction of each drug to the gene expression perturbations caused by the prostate cancer pathophenotype. In particular, we selected drugs whose signatures were negatively correlated with the prostate cancer signature, according to the CMap query tool [28,29,30], as able to have a potential treatment effect against genes that are a hallmark of prostate cancer phenotype (see Section 4).

In this case, the GSEA analysis highlighted that the candidate drugs with an in silico efficacy able to counteract the disease effect were those ones specifically predicted by the median metric with the highest percentage of 41%, followed by those ones specifically predicted by the mode and minimum metric with a percentage of 24% and 21%, respectively (Figure 4, Supplementary Table S4—second sheet).

## 3. Discussion

In this work, we proposed a comparison between different metrics used to compute the network-based proximity between the drug module and the disease module in the human interactome. In particular, we tested the standard minimum, maximum, mean, median, and mode metrics when applied to four diseases (i.e., liver cirrhosis, malignant breast neoplasm, schizophrenia, and prostate neoplasm), and we complemented the computation of the proximity value with a measure of statistical significance (*p*-value), obtained by applying a degree-randomization procedure. For each disease, the predicted compounds were those showing a statistically significant proximity value computed with each metric (*p*-value ≤ 0.05). No drugs were found statically significant by using the maximum metric. All the statistically significant drugs obtained with the other four metrics were then compared among each other in order to search for metric-specific drugs (Figure 5). From this comparison, our analysis highlighted some potentially interesting drugs specifically predicted by the other newly introduced metric, deepened in the next subsections.

### 3.1. Metric-Specific Off-Label Drugs: Mean

Among the drugs specifically predicted by using mean metric, amrinone emerged as a candidate repurposable drug for liver cirrhosis treatment (Figure 5a); whereas arzoxifene, bazedoxifene, ingenol mebutate, methyltestosterone, and conjugated estrogens emerged as candidate repurposable drugs for malignant neoplasm of breast (Figure 5b).

Amrinone is a type 3 pyridine phosphodiesterase inhibitor used for congestive heart failure treatment. However, some studies suggested that amrinone may play a significant role in the protection of liver against ischemia-reperfusion injury enhanced in cirrhotic patients, and that may be a pharmacological agent for safe and efficient liver surgery [31,32].

Arzoxifene is a selective estrogen receptor modulator (SERM) that antagonizes estrogen in mammary and uterine tissue and is investigated for treatment in breast cancer [33]. Several preclinical, phase I-II clinical studies showed that arzoxifene could be a promising endocrine therapy, demonstrating an ability to inhibit breast cancer cell growth in both in vitro and in vivo models, even if there is no evidence with phase III [34]. In addition, a network-meta analysis study showed that arzoxifene significantly reduced the risk of breast cancer [35]. Bazedoxifene is a SERM as well, which received approval alone or in combination with conjugated estrogens for treatment of moderate to severe vasomotor symptoms associated with menopause and prevention of postmenopausal osteoporosis.

Ingenol mebutate is a selective small molecule activator of protein kinase C approved for the topical treatment of actinic keratosis, but its application was also revealed as being effective for human and murine melanoma in mouse models, murine lung carcinoma, human prostate cancer, and human cervical carcinoma, and additional in vitro studies demonstrated that the drug could kill human breast cancer cells and T-leukemia cells [36,37].

Methyltestosterone is an anabolic steroid hormone used to treat men with a testosterone deficiency, but also used to treat other solid tumors, including breast cancer [38].

The conjugated estrogens are noncrystalline mixtures of purified female sex hormones obtained either by its isolation from the urine of pregnant mares or by synthetic generation from vegetal material and are indicated for the treatment of moderate to severe vasomotor symptoms due to menopause. In addition, the use of conjugated estrogens for a median of 5–9 years in postmenopausal women with hysterectomy was associated with a significant reduction in the incidence of invasive breast cancer based on a Women’s Health Initiative (WHI) randomized trial, where, with the estrogen use, a significant reduction was observed in breast cancer-related mortality and all-cause mortality after breast cancer diagnosis [39].

### 3.2. Metric-Specific Off-Label Drugs: Median

Among the drugs specifically predicted by using median metric, nesiritide was found as a candidate repurposable drug for liver cirrhosis (Figure 5a); while moclobemide and vinblatine were predicted for prostate neoplasm treatment (Figure 5d) and both were also confirmed by the GSEA analysis as they could counteract the gene expression perturbations caused by the prostate adenocarcinoma pathophenotype (Supplementary Table S4).

Nesiritide is a 32 amino acid recombinant human B-type natriuretic peptide used for the intravenous treatment of patients with acutely decompensated congestive heart failure who have dyspnea at rest or with minimal activity [40,41]. Although there are no clinical trials available, the mutual interaction between the heart and the liver dysfunctions has been investigated [42].

Moclobemide is a reversible monoamine oxidase inhibitor (MAO-I) selective for isoform A used to treat major depressive disorder. Recent reports indicated that high activity of MAO isozymes was associated with many neurodegenerative disorders, and showed elevated levels in several cancer types, including prostate cancers, and thus antidepressant MAO-Is could show anti-prostate cancer properties [43].

Vinblastine is a vinca alkaloid antineoplastic agent, with antitumor activity, targeting the microtubules of tumor cells, commonly applied for the treatment of several solid tumors and cancers, including breast cancer, testicular cancer, ovarian cancer, gastric cancer, and lung cancer, neuroblastoma, Hodgkin’s and non-Hodgkin’s lymphomas, and osteosarcoma [44,45,46].

### 3.3. Metric-Specific Off-Label Drugs: Mode

For what concerns drugs specifically predicted by using mode metric, we pointed out procarbazine as candidate repurposable for both malignant neoplasm of breast (Figure 5b) and prostate (Figure 5d); triamterene for treatment of schizophrenia (Figure 5c); and pargyline for prostate neoplasm (Figure 5d).

Procarbazine is an antineoplastic in the class of alkylating agents, which stop tumor growth by cross-linking guanine bases in DNA double-helix strands—directly attacking DNA [47]. It is primarily used in combination with mechlorethamine, vincristine, and prednisone for the treatment of stage III and stage IV Hodgkin’s disease, but it is also a type of chemotherapy drug in clinical trials for the treatment of other forms of cancers, including brain and central nervous system tumors [48].

Triamterene is a potassium-sparing diuretic that is indicated for the treatment of edema associated with congestive heart failure, cirrhosis of the liver, and nephrotic syndrome; also in steroid-induced edema, idiopathic edema, and edema due to secondary hyperaldosteronism [49]. Triamterene allows the maintenance of potassium balance, and hypokalemia is an identifiable, clinically important, and often overlooked condition in psychiatric patients [50].

Pargyline belongs to the monoamine oxidase inhibitors class with antihypertensive properties, thus it is indicated for the treatment of moderate to severe hypertension. However, it has been shown that in human prostate carcinoma cells, the proliferation of cells exposed to pargyline decreased in a dose- and time-dependent manner, the treatment with pargyline significantly induced cell cycle arrest at the G1 phase compared to the control samples, and also induced an increase in the cell death rate by promoting apoptosis [51].

## 4. Materials and Methods

### 4.1. Human Protein–Protein Interactome

The human protein–protein interactome was downloaded from Cheng and co-authors [17], where the authors merged their own systematic human protein–protein interactome and 15 commonly used databases with several types of experimental evidence (e.g., binary PPIs from 3-dimensional protein structures; Y2H, and/or literature-derived low-throughput experiments; signaling networks from literature-derived low-throughput experiments; kinase-substrate interactions from literature-derived low-throughput and high-throughput experiments; literature-curated PPIs identified by affinity purification followed by mass spectrometry). This version of the human interactome was composed of 217,160 protein–protein interactions (edges or links) connecting 15,970 unique proteins (nodes).

### 4.2. Disease-Gene Associations

Disease-associated genes were downloaded from DisGeNET [25], which is one of the largest publicly available collections of genes and variants associated with human diseases coming from GWAS, animal models, or scientific literature. The updated version of DisGeNET (v7.0) collects 1,134,942 gene-disease associations, between 21,671 genes and 30,170 diseases, disorders, traits, and clinical or abnormal human phenotypes. Among them, we selected a panel of 4 diseases of interest with their associated genes (Supplementary Table S1).

### 4.3. Drug-Target Interactions and Drug Medical Indications

Drug-target interactions were acquired from DrugBank [26], which is a comprehensive, freely accessible, online database containing information on drugs and drug targets. The updated version of DrugBank (version 5.1.6, released 22 April 2020) contains 13,563 drug entries, including 2627 approved small molecule drugs, 1373 approved biologics (proteins, peptides, vaccines, and allergenics), 131 nutraceuticals, and over 6370 experimental drugs. For our analysis, we selected a total of 1222 FDA-approved drugs with at least 2 annotated targets (Supplementary Table S1). The target Uniprot IDs were mapped to Entrez gene IDs by using BioMart—Ensembl tool (https://www.ensembl.org/, accessed on 27 March 2022).

The known drug medical indications were obtained from Therapeutic Target Database (TTD) [27], whose last version was released on 11 November 2019.

### 4.4. The Network-Based Proximity Measure

In order to investigate the extent to which the disease and drug modules were close in the human interactome, we used the standard network-based proximity measure defined in [17] as:

(1) $$p\left(T,S\right)\;=\frac{1}{\left\|T\right\|}\sum_{t\epsilon T}\underset{s\epsilon S}{\min\;}d(t,s)$$

**Figure 6:**
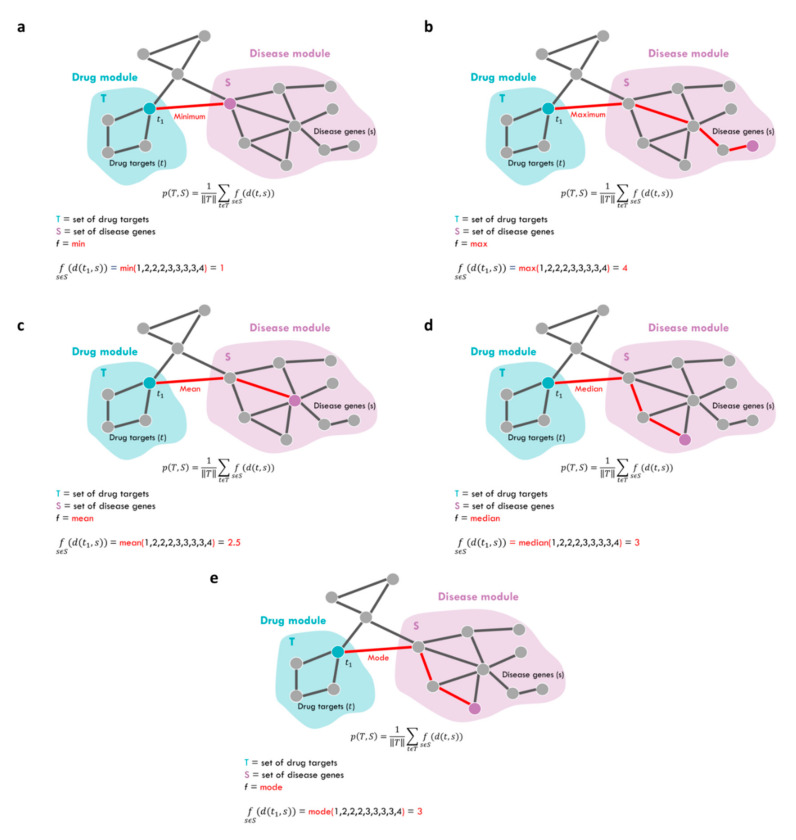
**Network-based proximity measures.** Schematic representation of the proximity measures computed between target proteins *t* of drug module *T* and disease genes *s* of disease module *S* according to five different metrics (**a**–**e**) described by Equations (1)–(5).

**Figure 1:**
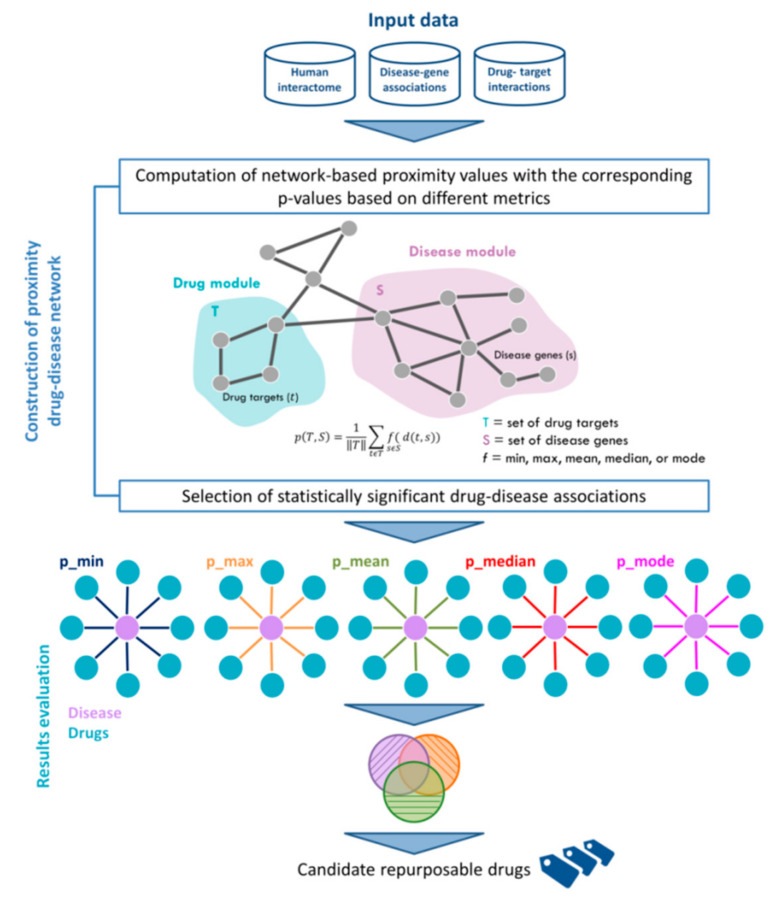
**Workflow of the analysis.** Input data are the human interactome network, the disease-gene associations from DisGeNET and the drug-targets interactions from DrugBank. The proximity measure between drug-targets and disease genes is computed by using five different metrics, including the standard minimum and the other here-proposed ones (i.e., maximum, mean, median, mode). The resulting candidate drugs are then compared among each metric, and metric-specific drugs are then discussed.

**Figure 2:**
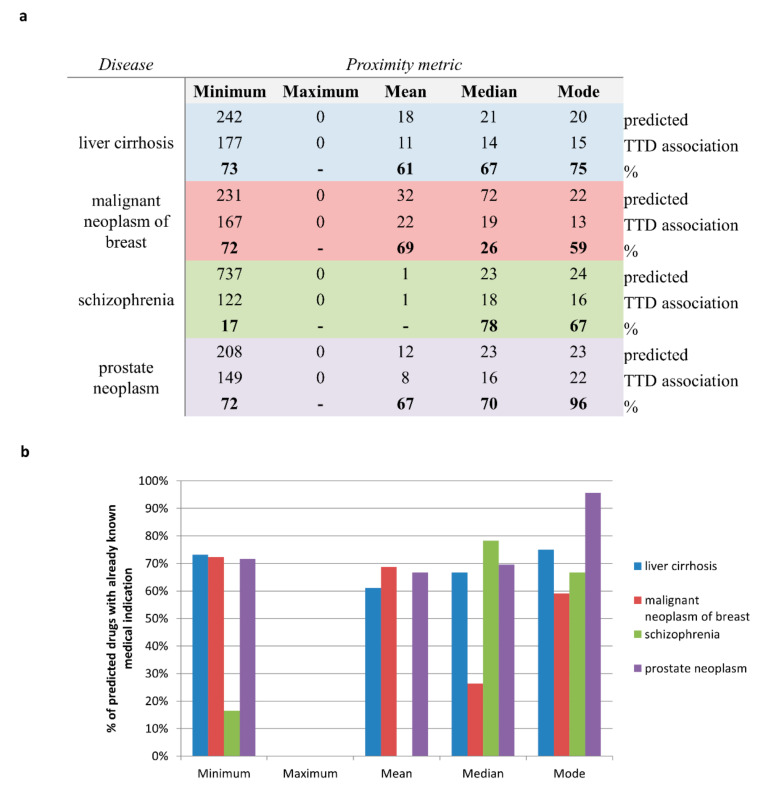
**Number of off-label predicted drugs according the five different metrics for each analyzed disease.** (**a**) The table reports, for each disease and each metric, the total number of predicted drugs, the number of predicted drugs that already have a known medical indication according to TTD database, and their ratio in terms of percentage (appearing in bold). (**b**) The bar plot shows the percentage of predicted drugs with already known medical indications grouped by metric for each disease reported in the legend. Only the metric predicting a total number of drugs greater than five for a specific disease are plotted.

**Figure 3:**
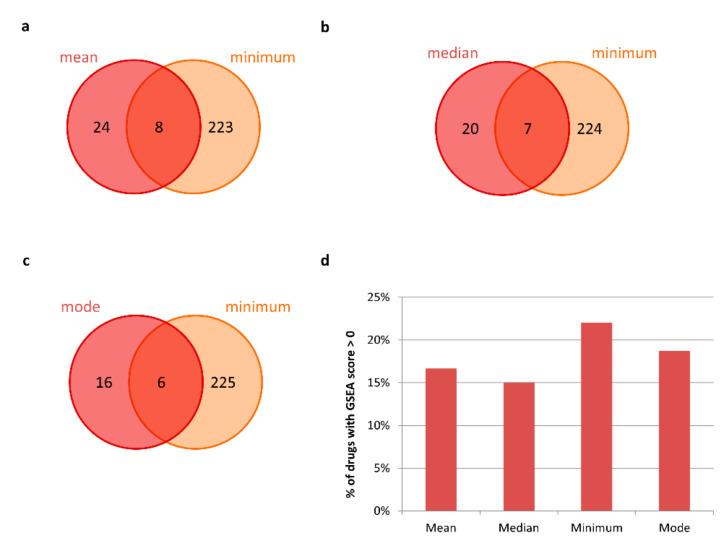
**In silico efficacy of candidate repurposable drugs for malignant breast neoplasm.** (**a**–**c**) Venn diagrams of the candidate drugs predicted by using mean, median, mode metrics with respect to the standard minimum one for malignant breast neoplasm treatment. (**d**) Bar plot showing the percentage of metric-specific candidate drugs that have a GSEA score greater than zero.

**Figure 4:**
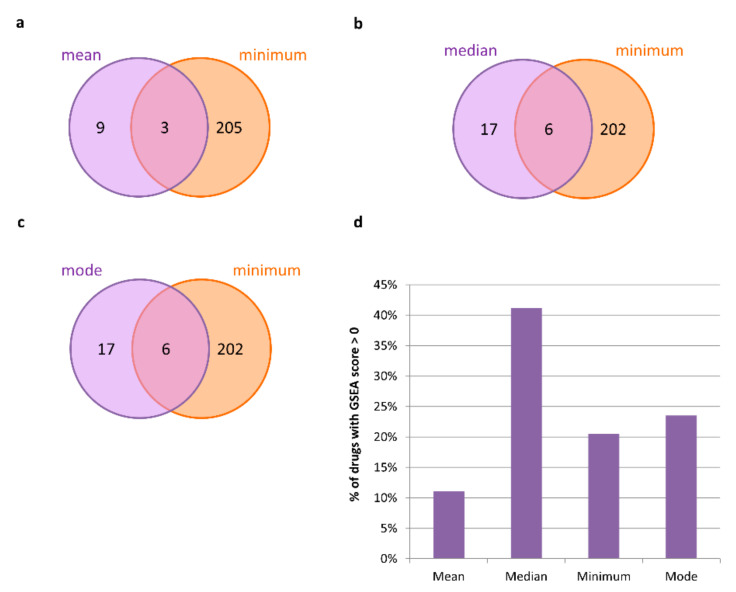
**In silico efficacy of candidate repurposable drugs for prostate neoplasm.** (**a**–**c**) Venn diagrams of the candidate drugs predicted by using mean, median, mode metrics with respect to the standard minimum one for prostate neoplasm treatment. (**d**) Bar plot showing the percentage of metric-specific candidate drugs that have a GSEA score greater than zero.

**Figure 5:**
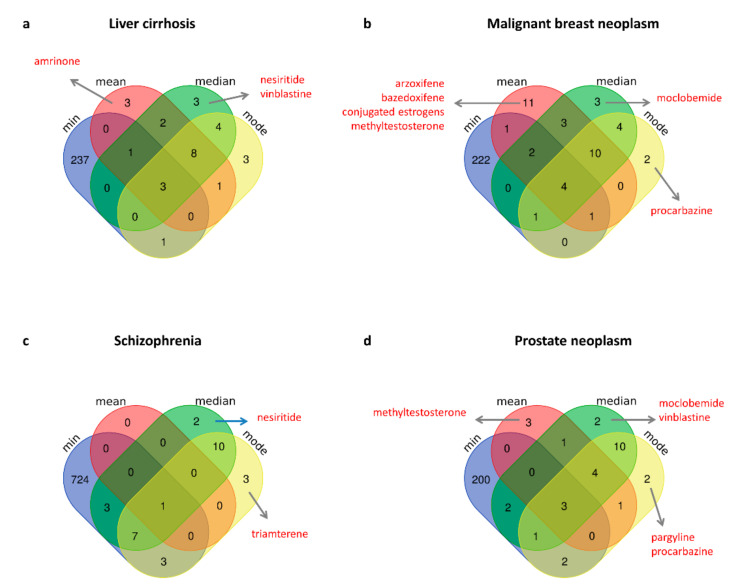
Venn diagram of the predicted repurposable drugs for each disease (**a**–**d**) according to the different exploited metrics. Metric-specific drugs with an already known relevant medical indication according to the TTD database and discussed in the text are highlighted in red.

Which represents the average of the shortest path length *d* between drug targets *t* in the drug module *T* and the nearest disease genes *s* in the disease module *S*. We computed the proximity measure by also using the other 4 different metrics (i.e., maximum, mean, median, and mode) to summarize the distances between drug module and disease module, defined as follows (Figure 6):

(2) $$p_{max}\left(T,S\right)\;=\frac{1}{\left\|T\right\|}\sum_{t\epsilon T}\underset{s\epsilon S}{\max\;}d(t,s)$$

(3) $$p_{mean}\left(T,S\right)\;=\frac{1}{\left\|T\right\|}\sum_{t\epsilon T}\underset{s\epsilon S}{mean\;}d(t,s)$$

(4) $$p_{median}\left(T,S\right)\;=\frac{1}{\left\|T\right\|}\sum_{t\epsilon T}\underset{s\epsilon S}{median\;}d(t,s)$$

(5) $$p_{mode}\left(T,S\right)\;=\frac{1}{\left\|T\right\|}\sum_{t\epsilon T}\underset{s\epsilon S}{mode\;}d(t,s)$$

To evaluate the statistical significance of each observed network proximity value between the 2 modules *T* and *S*, we built a reference distance distribution corresponding to the expected distance between 2 randomly selected groups of proteins with the same size and degree distribution of the original sets of disease proteins and drug targets in the human interactome. This procedure was repeated 1000 times, and the z statistics, together with the corresponding *p*-value, was computed by using the mean and the standard deviation of the reference distance distribution. We expected a *p*-value ≤ 0.05 for proximal drug and disease modules.

### 4.5. Gene Set Enrichment Analysis

In order to test whether the candidate repurposable drugs for malignant breast and prostate neoplasm predicted by applying the different metrics for proximity computation could counteract the gene expression perturbations caused by the pathophenotype (i.e., if they could up-regulate genes down-regulated by the disease or vice versa), we performed a gene set enrichment analysis (GSEA). We first collected from The Cancer Genome Atlas (TCGA) [52] RNA-sequencing expression data of (i) breast invasive carcinoma from 113 patients and (ii) prostate adenocarcinoma from 52 patients, for which the complete sets of tumor and matched-normal profiles were available. RNA sequencing data corresponded to normalized expression data from RNASeq Version 2 created using MapSplice to conduct the alignment and RSEM to perform the quantification and normalization. For each disease, data were processed by applying a logarithmic (log2) transformation of the expression values, and by conducting a preprocessing analysis via the computation of the Inter Quartile Range (IQR) for each gene. IQR is a measure of data variability around the median that is equal to the difference between the 75th and 25th percentiles of the data distribution. Those genes with an IQR value smaller than the 10th percentile of the IQR distribution (corresponding to those genes less scattered around the median) were filtered out. Then, we performed a paired *t*-student test, and we adjusted the obtained *p*-values for multiple hypotheses testing by using the Benjamini–Hochberg procedure [53]. In order to select statistically significant differentially expressed genes, we set a threshold of 0.01 on the adjusted *p*-values. We used the so-defined lists of differentially expressed genes of breast cancer and prostate cancer as *disease* signatures.

Then, we queried the Connectivity Map (CMap) database that collects high-throughput reduced representation gene expression data obtained by using an L1000 assay [28,54]. The L1000 profiling was performed in a variety of drug-treated human cell lines for which there were well-established culture and treatment protocols. Thus, the CMap database of cellular signatures cataloged transcriptional responses of human cells to chemical and genetic perturbation. A total of 27,927 perturbagens were profiled in a core set of 9 cell lines to produce 476,251 expression signatures. In particular, we selected the drugs-treated cells lines available from the CMap database for human breast adenocarcinoma (i.e., MCF7 cell line) and for human prostate adenocarcinoma (i.e., PC3 cell line) and we used them as *drug* signatures.

By exploiting the CMap query tool, we evaluated the treatment effects of each drug signature (i.e., differentially expressed genes of drugs-treated human cell lines included in CMap database) on each disease signature (i.e., differentially expressed genes of breast cancer or prostate cancer) [54]. The disease and the drug signatures were ranked by fold-change, and then CMap computed an enrichment score (ES) that measured if the effect of the drug could counteract the effect of the disease (ES < 0), or not (ES > 0) [28,29]. The idea behind this was the following: 1 ordered disease signature was compared to 1 ordered drug signature to determine whether the highest up-regulated (down-regulated) gene in the disease signature was near the bottom (top) of the drug signature. This would mean that the drug and disease have complementary expression profiles (ES < 0), and the drug might be a possible treatment option for the disease of interest. Details on the computation of this score were provided in [28,29,30]. In particular, a selected repurposing candidate drug was considered to have a potential treatment effect against the analyzed disease if the drug signature was negatively correlated with the disease signature. We stated that drugs and disease were negatively correlated if the corresponding ES was negative, and we assigned a score equal to 1 to that drug for that disease signature.

## 5. Conclusions

In this study, we implemented a computational analysis for identifying new uses for approved drugs that were outside the scope of the original medical indication. Specifically, we exploited the well-established network-based drug-disease proximity measure proposed in [17] by using four different metrics (i.e., maximum, mean, median, mode), instead of the standard minimum to compute the distance between the drug module and the disease module in the human interactome. We complemented the computation of the proximity value of each metric with a measure of statistical significance (*p*-value) corresponding to the z-score normalization of the proximity obtained by applying a degree-preserving randomization procedure. Thus, for each metric, the candidate proposed drugs as those ones showing a proximity value with a *p*-value ≤ 0.05. We then conducted a comparison study of the candidate drugs predicted with these here-implemented metrics with respect to those ones obtained by using the standard minimum when applied to four diseases of interest (i.e., liver cirrhosis, schizophrenia, malignant breast neoplasm, and prostate neoplasm).

One limitation of this analysis is its computational nature. However, in order to have a clue of the potential efficacy of the predicted repurposable molecules of each metric, we also complemented the study with an in silico validation by exploiting CMap database, which is a comprehensive collection of drug-treated cell lines (drug signature), and TCGA repository, which is a collection of gene expression profiles for healthy and sick patients. By computing the differentially expressed genes, we evaluated the effect of the disease on gene modulation (disease signature). Then, studying the correlation between the drug signature and disease signature, we evaluated those drugs that could potentially counteract the disease effect (i.e., negative correlation between drug signature and disease signature). Taken together, our findings confirmed that the original network-based proximity metric based on the minimum distance between drug and disease module is the most reliable tool for screening off-label drugs, but also some of the here-implemented metrics specifically highlighted some interesting drug candidates worthy of further investigation.

Yet, another limitation of this approach is that our procedure does not implement a method to estimate false positive values, and thus assigns a score to all compounds available from DrugBank.

## Figures and Tables

**Table 1:** Classification of breast cancer patients (pz) retrieved from TCGA based on ER, PR, HER receptors status.

**Receptor/Classification**	**Luminal A**	**Luminal B-Like**	**Luminal B-Like**	**Luminal B**	**HER2-Enriched**	**Triple Negative**
ER	positive	positive	positive	positive	negative	negative
PR	positive	positive	negative	negative	negative	negative
HER2	negative	positive	positive	negative	positive	negative
**number of pz**	38	15	2	7	4	11
	**less aggressive (81%)**				**more aggressive (19%)**	

## Data Availability

All relevant data are within the manuscript and its Supporting Information files; R code is freely available at https://github.com/giuliafiscon/GeneralizedProximity.git (accessed on 27 March 2022).

## Supplementary Materials

The following supporting information can be downloaded at: https://www.mdpi.com/article/10.3390/ijms23073703/s1.

## References

[1] Pushpakom S., Iorio F., Eyers P.A., Escott K.J., Hopper S., Wells A., Doig A., Guilliams T., Latimer J., McNamee C. (2019). Drug repurposing: Progress, challenges and recommendations. Nat. Rev. Drug Discov..

[2] Sonawane A.R., Weiss S.T., Glass K., Sharma A. (2019). Network Medicine in the Age of Biomedical Big Data. Front. Genet..

[3] Barabási A.-L., Gulbahce N., Loscalzo J. (2011). Network medicine: A network-based approach to human disease. Nat. Rev. Genet..

[4] Caldera M., Buphamalai P., Müller F., Menche J. (2017). Interactome-based approaches to human disease. Curr. Opin. Syst. Biol..

[5] Silverman E.K., Schmidt H.H.H.W., Anastasiadou E., Altucci L., Angelini M., Basimon L., Balligand J.-L., Benincasa G., Capasso G., Conte F. (2020). Molecular networks in Network Medicine: Development and applications. WIREs Syst. Biol. Med..

[6] Paci P., Fiscon G., Conte F., Wang R.-S., Farina L., Loscalzo J. (2021). Gene co-expression in the interactome: Moving from correlation toward causation via an integrated approach to disease module discovery. NPJ Syst. Biol. Appl..

[7] Paci P., Fiscon G., Conte F., Licursi V., Morrow J., Hersh C., Cho M., Castaldi P., Glass K., Silverman E.K. (2020). Integrated transcriptomic correlation network analysis identifies COPD molecular determinants. Sci. Rep..

[8] Panebianco V., Pecoraro M., Fiscon G., Paci P., Farina L., Catalano C. (2020). Prostate cancer screening research can benefit from network medicine: An emerging awareness. NPJ Syst. Biol. Appl..

[9] Conte F., Fiscon G., Licursi V., Bizzarri D., D’Antò T., Farina L., Paci P. (2020). A paradigm shift in medicine: A comprehensive review of network-based approaches. Biochim. Biophys. Acta BBA-Gene Regul. Mech..

[10] Tieri P., Farina L., Petti M., Ranganathan S., Gribskov M., Nakai K. (2019). Network Inference and Reconstruction in Bioinformatics. Encyclopedia of Bioinformatics and Computational Biology.

[11] Fiscon G., Pegoraro S., Conte F., Manfioletti G., Paci P. (2021). Gene network analysis using SWIM reveals interplay between the transcription factor-encoding genes HMGA1, FOXM1, and MYBL2 in triple-negative breast cancer. FEBS Lett..

[12] Falcone R., Conte F., Fiscon G., Pecce V., Sponziello M., Durante C., Farina L., Filetti S., Paci P., Verrienti A. (2019). BRAFV600E-mutant cancers display a variety of networks by SWIM analysis: Prediction of vemurafenib clinical response. Endocrine.

[13] Paci P., Colombo T., Fiscon G., Gurtner A., Pavesi G., Farina L. (2017). SWIM: A computational tool to unveiling crucial nodes in complex biological networks. Sci. Rep..

[14] Fiscon G., Conte F., Licursi V., Nasi S., Paci P. (2018). Computational identification of specific genes for glioblastoma stem-like cells identity. Sci. Rep..

[15] Fiscon G., Conte F., Paci P. (2018). SWIM tool application to expression data of glioblastoma stem-like cell lines, corresponding primary tumors and conventional glioma cell lines. BMC Bioinform..

[16] Grimaldi A.M., Conte F., Pane K., Fiscon G., Mirabelli P., Baselice S., Giannatiempo R., Messina F., Franzese M., Salvatore M. (2020). The New Paradigm of Network Medicine to Analyze Breast Cancer Phenotypes. Int. J. Mol. Sci..

[17] Cheng F., Desai R.J., Handy D.E., Wang R., Schneeweiss S., Barabásiet A.-L., Loscalzoal J. (2018). Network-based approach to prediction and population-based validation of in silico drug repurposing. Nat. Commun..

[18] Cheng F., Liu C., Jiang J., Lu W., Liu G., Zhou W., Huang J., Tang Y. (2012). Prediction of Drug-Target Interactions and Drug Repositioning via Network-Based Inference. PLoS Comput. Biol..

[19] Zhou Y., Hou Y., Shen J., Huang Y., Martin W., Cheng F. (2020). Network-based drug repurposing for novel coronavirus 2019-nCoV/SARS-CoV-2. Cell. Discov..

[20] Fang J., Zhang P., Wang Q., Zhou Y., Chiang C.-W., Chen R., Zhang B., Li B., Lewis S.J., Pieper A.A. (2020). Network-based Translation of GWAS Findings to Pathobiology and Drug Repurposing for Alzheimer’s Disease. medRxiv.

[21] Gysi D.M., Valle Í.D., Zitnik M. (2020). Network Medicine Framework for Identifying Drug Repurposing Opportunities for COVID-19. https://arxiv.org/abs/2004.07229.

[22] Fiscon G., Paci P. (2021). SAveRUNNER: An R-based tool for drug repurposing. BMC Bioinform..

[23] Fiscon G., Conte F., Farina L., Paci P. (2021). SAveRUNNER: A network-based algorithm for drug repurposing and its application to COVID-19. PLOS Comput. Biol..

[24] Fiscon G., Conte F., Amadio S., Volonté C., Paci P. (2021). Drug repurposing: A network-based approach to Amyotrophic Lateral Sclerosis. Neurotherapuetics.

[25] Piñero J., Ramírez-Anguita J.M., Saüch-Pitarch J., Ronzano F., Centeno E., Sanz F., Furlong F.I. (2020). The DisGeNET knowledge platform for disease genomics: 2019 update. Nucleic Acids. Res..

[26] Wishart D.S., Feunang Y.D., Guo A.C., Lo E.J., Marcu A., Grant J.R., Sajed T., Johnson D., Li C., Sayeeda Z. (2018). DrugBank 5.0: A major update to the DrugBank database for 2018. Nucleic Acids. Res..

[27] Wang Y., Zhang S., Li F., Zhou Y., Zhang Y., Wang Z., Zhang R., Zhu J., Ren Y., Tan Y. (2020). Therapeutic target database 2020: Enriched resource for facilitating research and early development of targeted therapeutics. Nucleic Acids. Res..

[28] Lamb J., Crawford E.D., Peck D., Modell J.W., Blat I.C., Wrobel M.J., Lerner J., Brunet J.-P., Subramanian A., Ross K.M. (2006). The Connectivity Map: Using gene-expression signatures to connect small molecules, genes, and disease. Science.

[29] Sirota M., Dudley J.T., Kim J., Chiang A.P., Morgan A.A., Sweet-Cordero A., Sage J., Butte A.J. (2011). Discovery and preclinical validation of drug indications using compendia of public gene expression data. Sci. Transl. Med..

[30] Subramanian A., Tamayo P., Mootha V.K., Ebert B.L., Gillette M.A., Paulovich A., Pomeroy S.L., Golub T.R., Lander E.S., Mesirov J.P. (2005). Gene set enrichment analysis: A knowledge-based approach for interpreting genome-wide expression profiles. Proc. Natl. Acad. Sci. USA.

[31] Kucuk C., Akcan A., Akyýldýz H., Akgun H., Muhtaroglu S., Sozuer E. (2009). Effects of amrinone in an experimental model of hepatic ischemia-reperfusion injury. J. Surg. Res..

[32] Orii R., Sugawara Y., Hayashida M., Yamada Y., Chang K., Takayama T., Makuuchi M., Hanaoka K. (2000). Effects of amrinone on ischaemia-reperfusion injury in cirrhotic patients undergoing hepatectomy: A comparative study with prostaglandin E1. Br. J. Anaesth.

[33] Arzoxifene. https://go.drugbank.com/drugs/DB06249.

[34] Jackson L.R., Cheung K.L., Buzdar A.U., Robertson J.F.R. (2007). Arzoxifene: The evidence for its development in the management of breast cancer. Core. Evid..

[35] Mocellin S., Pilati P., Briarava M., Nitti D. (2016). Breast Cancer Chemoprevention: A Network Meta-Analysis of Randomized Controlled Trials. JNCI J. Natl. Cancer. Inst.

[36] Ersvaer E., Kittang A.O., Hampson P., Sand K., Gjertsen B.T., Lord J.M., Bruserud Y. (2010). The Protein Kinase C Agonist PEP005 (Ingenol 3-Angelate) in the Treatment of Human Cancer: A Balance between Efficacy and Toxicity. Toxins.

[37] Mansuy M., Nikkels-Tassoudji N., Arrese J.E., Rorive A., Nikkels A.F. (2014). Recurrent In Situ Melanoma Successfully Treated with Ingenol Mebutate. Dermatol. Ther..

[38] Methyltestosterone. https://go.drugbank.com/drugs/DB06710.

[39] Anderson G.L., Chlebowski R.T., Aragaki A.K., Kuller P.L.H., Manson P.J.E., Gass P.M., Bluhm E., Connelly P.S., Hubbell P.F.A., Lane P.D. (2012). Conjugated equine oestrogen and breast cancer incidence and mortality in postmenopausal women with hysterectomy: Extended follow-up of the Women’s Health Initiative randomised placebo-controlled trial. Lancet Oncol..

[40] Keating G.M., Goa K.L. (2003). Nesiritide: A review of its use in acute decompensated heart failure. Drugs.

[41] O’Connor C.M., Starling R.C., Hernandez A.F., Armstrong P.W., Dickstein K., Hasselblad V., Heizer G.M., Komajda M., Massie B.M., McMurray J.J.V. (2011). Effect of Nesiritide in Patients with Acute Decompensated Heart Failure. N. Engl. J. Med..

[42] El Hadi H., Di Vincenzo A., Vettor R., Rossato M. (2020). Relationship between Heart Disease and Liver Disease: A Two-Way Street. Cells.

[43] Zarmouh N.O., Messeha S.S., Mateeva N., Gangapuram M., Flowers K., Eyunni S.V.K., Zhang W., Redda K.K., Soliman K.F.A. (2020). The Antiproliferative Effects of Flavonoid MAO Inhibitors on Prostate Cancer Cells. Molecules.

[44] Coderch C., Morreale A., Gago F. (2012). Tubulin-based structure-affinity relationships for antimitotic Vinca alkaloids. Anticancer. Agents. Med. Chem..

[45] Koontz M.Z., Horning S.J., Balise R., Greenberg P.L., Rosenberg S.A., Hoppe R.T., Advani R.H. (2013). Risk of therapy-related secondary leukemia in Hodgkin lymphoma: The Stanford University experience over three generations of clinical trials. J. Clin. Oncol. J. Am. Soc. Clin. Oncol..

[46] Vinblastine. https://go.drugbank.com/drugs/DB00570.

[47] Kyrtopoulos S.A., Anderson L.M., Chhabra S.K., Souliots V.L., Pletsa V., Valavanis C., Georgladis P. (1997). DNA adducts and the mechanism of carcinogenesis and cytotoxicity of methylating agents of environmental and clinical significance. Cancer Detect. Prev..

[48] Procarbazine. https://go.drugbank.com/drugs/DB01168.

[49] Triamterene. https://go.drugbank.com/drugs/DB00384.

[50] Hong E. (2016). Hypokalemia and Psychosis: A Forgotten Association. Am. J. Psychiatry Resid J..

[51] Lee H.T., Choi M.R., Doh M.S., Jung K.H., Chai Y.G. (2013). Effects of the monoamine oxidase inhibitors pargyline and tranylcypromine on cellular proliferation in human prostate cancer cells. Oncol. Rep..

[52] Tomczak K., Czerwinska P., Wiznerowicz M. (2015). The Cancer Genome Atlas (TCGA): An immeasurable source of knowledge. Contemp. Oncol. Pozn..

[53] Benjamini Y., Hochberg Y. (1995). Controlling the false discovery rate: A practical and powerful approach to multiple testing. J. R. Stat. Soc. Ser. B Methodol..

[54] Subramanian A., Narayan R., Corsello S.M., Peck D.D., Natoli T.E., Lu X., Gould J., Davis J.F., Tubelli A.A., Asiedu J.K. (2017). A Next Generation Connectivity Map: L1000 Platform and the First 1,000,000 Profiles. Cell.

